# Impact of New Clinical Policies during the COVID-19 Pandemic on Clinical Incidents and Complaints at a UK Teaching Hospital

**DOI:** 10.3390/ijerph18083979

**Published:** 2021-04-09

**Authors:** William Atiomo, Peter Weir, Lucy Kean

**Affiliations:** 1 Division of Family Health, Nottingham University Hospitals NHS Trust, Queen’s Medical Centre, Derby Road, Nottingham NG7 2UH, UK; lucy.kean@nuh.nhs.uk; 2 School of Medicine, University of Nottingham, B Floor, Medical School, Queen’s Medical Centre, Nottingham NG7 2UH, UK; mzypaw@exmail.nottingham.ac.uk

**Keywords:** COVID, Corona, incidents, complaints, obstetrics, gynaecology, paediatrics, child, quality, safety

## Abstract

Background: To investigate any associations between new clinical policies implemented because of the COVID-19 pandemic and harm to patients. Methods: Retrospective data collection of incidents and complaints reported through Datix^®^, and the Patient Advice and Liaison Service (PALS), respectively. The setting was the Family Health division in a University teaching hospital in the UK. Primary and secondary outcome measures included: the proportion of incidents reported on Datix^®^ from 23 March 2020 to 29 May 2020, compared to the period from 23 March 2019 to 29 May 2019. COVID-19 related incidents and complaints and association with newly published guidelines or pathways from 23 March 2020 to 29 May 2020 were investigated. Results: There was no significant difference in the proportion of overall patient activity resulting in incidents reported on Datix in 2020 (2.08%) compared to 2019 (2.09%), with 98% resulting in no/low harm in 2020. Three incident categories had increases in relative proportions of incidents including the terms “COVID” or “Corona” compared to incidents that did not: “Child death”, “delay/failure to treatment and procedure” and “information governance”. One of the child deaths was a miscarriage and we were unable to link the second child death to a change in clinical policy at this stage. We were only able to link two COVID-19 associated incidents with a pathway or procedural change (one to the Children’s Emergency Department admission pathway and the second to the introduction of virtual antenatal clinics). Eighteen complaints related to COVID-19 were logged. However, at this stage, we are unable to link any of these to a published change in clinical policy. Conclusions: New policies introduced in the division, during the COVID-19 pandemic were associated with similar rates of clinical incidents, when compared with the previous year. There were only two COVID-19-related incidents clearly related to a change in pathways and procedures. Continued surveillance and improved metrics for monitoring the impact of changes to pathways and procedures should be sought with the sustained presence of COVID-19 in clinical areas.

## 1. Introduction

The Coronavirus disease 2019 (COVID-19) [1] pandemic resulted in an unprecedented change in global health care delivery. As of 2 March 2021, 115,198,775 cases had been reported globally with unfortunately 2,554,564 deaths. The corresponding figures in the UK, were, 4,188,400 cases and 123,296 deaths [2]. In response to the pandemic, in a televised address, the British prime minister, The Rt Hon., Boris Johnson MP, announced a UK-wide partial lockdown, to contain the spread of the virus. The British public were instructed that they must stay at home, except for certain “very limited purposes”—shopping for basic necessities; for “one form of exercise a day”; for any medical need; and to travel to and from work when “absolutely necessary” [3].

Several measures were also rapidly introduced by hospitals in the United Kingdom to cope with the additional potential burden of the pandemic. National Health Service (NHS) England for example announced that all non-urgent treatment would be postponed from 15 April to free up to 30,000 beds with many hospitals introducing virtual consultations [4] to reduce the number of face to face consultations to minimize the risks of transmitting the COVID-19 infection and ensure patient safety.

Locally, at our hospital, Nottingham University Hospital (NUH) NHS Trust, in response to the COVID-19 pandemic, a series of new and/or revised clinical pathways and procedures were rapidly implemented, the majority of which were still in effect as of August 2020. Prior to this study, there had however been no study investigating the impact of these changes to health service delivery on patient safety. One pre-print (pre-peer review) study [5] from the UK found that error reporting amongst staff measured from Datix^®^ dropped after the COVID-19 pandemic, but they did not measure actual clinical incidents reported. Another study [6], found that institutional births were reduced, with an increased risk of preterm births, still births and neonatal mortality during lockdown, but the study did not directly investigate the impact of changes in hospital policy on patient safety.

This study therefore aimed to investigate any association between new clinical policies introduced as a result of the COVID-19 and harm to patients within the Family Health (FH) division at NUH. Specifically, we were investigating whether any new policies or procedures introduced during the initial phase of the COVID-19 pandemic led to an increase in the number of clinical incidents reported, when compared to a similar period in the previous year, as we prepared to restore our departmental services to treat an increased number of patients as the COVID-19 lockdown restrictions were gradually lifted. This was to inform how possible shortcomings could be addressed to ensure safe practice in the continued presence of COVID-19. The Parliamentary and Health Service Ombudsman paused their work on existing NHS complaints and acceptance of new health complaints from 26 March 2020 to 30 June 2020, and this study also provided an opportunity to find out if this had an impact on incident reporting rates in our hospital during the COVID-19 pandemic [7].

## 2. Methods

### 2.1. Study Design

This was a retrospective study carried out in June 2020, with further data analysis in February and March 2021. The focus on the FH division which covers Obstetrics, Gynaecology, Paediatrics (Children’s Hospital), Clinical Genetics and Sexual Health was because two of the authors were in the senior management team of the division and felt it was very important to inform their decision making on objective data on patient safety.

### 2.2. Ethical Considerations

Ethics committee approval was not thought to be required for the study because it was a desk-based service review, that did not involve any patient contact. The Medical Research Council (MRC) Regulatory Support Centre/the UK NHS Health Research Authority (HRA) online decision support tool (http://www.hra-decisiontools.org.uk/research/ accessed on 2 November 2020), also, did not class the study as research.

### 2.3. Study Subjects and Data Collection

Data covering the time period 23 March 2020 to 29 May 2020 were collected. The date range was selected as it represented the first 9 weeks following the UK-wide lockdown, whilst preceding the restoration and recovery of clinical pathways in the NUH FH division.

Incidents and complaints in the division were reported by people in a range of roles, including medical and non-medical staff, patients, carers, parents, or guardians. Data on all incidents logged on the Datix^®^ software, and complaints registered with the Patient Advice and Liaison Service (PALS) were obtained. In addition, details of all procedural changes and revised protocols in this time period (23 March 2020 to 29 May 2020) were obtained. Data collection was carried out by the FH clinical effectiveness team, the NUH PALS team and staff in the data support unit.

### 2.4. Outcome Measures and Data Analysis

All retrieved data were transferred to a Microsoft Excel^®^ spreadsheet for analysis by one of the authors. The data were analysed as follows: A Datix^®^ and free text search for “covid” or “corona” was performed by two authors (WA and PW). Following this, any incidents identified as relating to “covid” or “corona” were read and linked to the title of a published new clinical policy in response to the COVID-19 pandemic by one author (WA). Data from 23 March 2020 to 29 May 2020, were compared with data from 23 March 2019 to 29 May 2019 (similar period one year earlier). Data were then summarized as proportions.

In order to investigate the possible impact of selection bias on the results, data on the population and patient characteristics (including age, gender, length of stay in hospital of all admissions and numbers of new and follow up appointments) of all patients seen in the NUH FH division were retrospectively requested from our divisional data analysts by WA in February 2021. Data covering the study periods from 23 March 2020 to 29 May 2020 and 23 March 2019 to 29 May 2019 (a similar period one year earlier) were requested.

The significance of statistical comparisons between categorical variables was calculated using the chi-squared test and for continuous variables, the Student *t*-test. A *p* value of less than 0.05 was considered significant for this purpose. Statistical analyses were done using a publicly available statistical package available at http://www.obg.cuhk.edu.hk/ResearchSupport/StatTools/Unpaired2Props_Pgm.php (last accessed on 2 March 2021).

The operational definition of “incident”, was a reported clinical incident on our hospital Datix^®^ system, “COVID-19 related incidents” were those clinical incidents reported on our hospital Datix^®^ system which included the words “COVID” or “Coronavirus” in the text of the incident report and “COVID-19 related complaints” were the details of the complaints provided to us by the PALS team when we requested a list of the complaints registered with them that were related to COVID-19. The operational definition of the degree (no, low, moderate, severe) of harm in association with the clinical incident reported in our study, was “no”, where no harm had been caused, “low”, where minimal harm had been caused, “moderate”, where short term harm had been caused and “severe”, where permanent or long-term harm had been caused. This information was required as part of the Datix^®^ reporting system and provided by the member of staff inputting the information on the clinical incident into the Datix^®^ system at the time the incident was reported.

### 2.5. Patient and Public Involvement

No patients or members of the public were involved in this study.

## 3. Results

### 3.1. Patient Activity Data

There were 7138 inpatient episodes and 25,099 outpatient attendances in the NUH FH division from 23 March 2020 to 29 May 2020 just after the COVID-19 lockdown. A total patient activity count of 32,237 was recorded. The numbers for 23 March 2019 to 29 May 2019, before the COVID-19 lockdown, were 9411 inpatient episodes and 30,027 outpatient episodes. A total activity count of 39,438 was recorded. This represented a 24%, 16% and 18% drop in inpatient episodes, outpatient attendances and total patient activity count, respectively, in the NUH FH division.

### 3.2. Incident Reporting Rates

There was no statistically significant difference in the proportion of incidents reported on Datix^®^ from 23 March 2020 to 29 May 2020, compared to the period from 23 March 2019 to 29 May 2019. Six hundred and seventy-two (672) (2.08% of overall patient activity) incidents were reported in 2020 compared to 826 (2.09% of overall patient activity) incidents reported in 2019. Of these, one serious untoward incident (0.0031% of the overall patient activity) was reported in 2020 and one serious untoward incident (0.0025% of the overall patient activity) was reported in 2019. The serious incident in 2020 involved a baby born in poor condition following a forceps delivery, whilst the serious incident in 2019 involved delayed recognition of jaundice in a 7-day-old baby.

Although the serious untoward incident in 2020 following the COVID-19 lockdown was still being investigated at the time of initial data analysis, there was no obvious indication to suggest that it arose because of a change in health care delivery pathway or procedure by the NUH FH division, introduced in response to the COVID-19 pandemic.

Most (656 out of 672 (98%)) of the incidents reported after the COVID-19 pandemic in 2020 resulted in no or low harm. The corresponding figures in 2019 were 821 out of 826 (99%). There was no statistically significant difference found in the proportion of incidents classed as resulting in low or no harm in 2020 compared with 2019. There was however a significant increase in the number of incidents reported as resulting in moderate harm in 2020 compared to 2019 (12 (1.79%) vs. 4 (0.48%)), *p* < 0.05.

### 3.3. COVID-19-Related Incidents and Association with Newly Published Guidelines or Pathways in NUH FH Division (23 March 2020 to 29 May 2020)

Twenty-eight (28) patients with diagnosed COVID-19 infection were admitted to the NUH FH division in the above period but no deaths were recorded. Fifty-six (56) new clinical policies were created across the division during the study period. Of these, 28 (50%) were in the children and young people’s (paediatrics) services, 13 (23%) in maternity, 9 (16%) in gynaecology, and 6 (11%) in sexual health.

**Figure 1:**
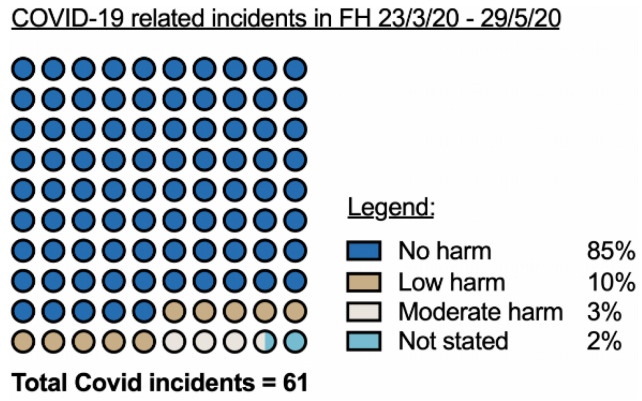
The degree of harm resulting from incidents deemed to be COVID-19-related in the period (23 March 2020 to 29 May 2020): no harm (*n* = 52), low harm (*n* = 6), moderate harm (*n* = 2), not stated (*n* = 1). FH = Family Health Division at Nottingham University Hospitals NHS Trust. UK.

Of the 672 incidents reported, 61 (9% of reported incidents) included the words “COVID” or “Coronavirus”. A total of 52 were classed as resulting in no harm to the patient, (85% of COVID-19-related incidents), 6 low harm (10% of COVID-19-related incidents), 2 moderate harm (3% of COVID-19 related incidents) and in 1 case the degree of harm (2% of COVID-19-related incidents) was not stated, which was the case of a child death (a young boy (in the age range 10–15 years old) who died from septic shock). Overall, 95% of all the 61 COVID-19-related incidents resulted in no or low harm (Figure 1). Three of the 61 COVID-19-related incidents involved patients infected directly with the virus.

Three incident categories (Table 1) had statistically significant increases in relative proportions of incidents including terms “COVID” or “Corona” compared to the set of incidents that did not include these terms: “Child death” (∆ (difference) 3%, *p* = 0.0472), “delay/failure to treatment and procedure” (∆10.3%, *p* = 0.0123) and “information governance” (∆8.4%, *p* = 0.003). With respect to the child deaths, there were two incidents reported after the COVID-19 pandemic between 23 March 2020 and 29 May 2020. We were unable to clearly link either of these two child deaths with a pathway or procedural change in FH. One child death was an inevitable miscarriage, and the second child death was a young boy (in the age range 10–15 years old) who died from septic shock. Three of the four incidents of aggression, violence, or harassment were consequences of Trust-wide changes to the policy relating to relatives visiting patients. At the stage of data analysis, without further detailed root cause analyses, we were only able to link two COVID-19 associated incidents with a pathway or procedural change in FH (one to the Children’s emergency department (ED) admission pathway and the second to the introduction of virtual antenatal clinics). These changes were introduced on 16 March 2020 and 30 March 2020, respectively, around the time of the first country-wide, UK COVID-19 lockdown, announced by the British Prime Minister, The Rt Hon., Boris Johnson MP, on 23 March 2020.

### 3.4. COVID-19-Related Complaints and Association with Newly Published Guidelines or Pathways in NUH FH Division (23 March 2020 to 29 May 2020)

In the same time period (23 March 2020 to 29 May 2020) 18 complaints related to COVID-19 were logged through PALS. However, at the stage of data analysis, without formal investigation of the complaints, we were unable to patently connect/link any of these to a published pathway or procedural change. The complaints were spread across four categories (Table 2). Six complaints regarding clinical treatment, five complaints regarding patient safety, four complaints regarding communication and three complaints regarding appointments were registered.

### 3.5. Population and Patient Characteristics NUH Family Health Division in 2019 Compared with 2020

With respect to the data retrospectively provided to us by the NUH FH data analysts, the latest date of admission on the data on hospital admissions for 2019 provided was 28 May 2019 and in 2020, 28 May 2020. Data analysis was therefore restricted to patients seen during the COVID19 pandemic from 23 March 2020 to 28 May 2020 and compared to patients seen in 2019 before the pandemic from 23 March 2019 to 28 May 2019 (similar period one year earlier). Table 3 shows the results. There was no significant difference in the proportion of patients of a male or female gender in both time periods. The mean age of patients admitted to hospital in 2020 was slightly higher compared to 2019 (18.9 years vs. 17.2 years, *p* < 0.0001) as was the mean age of patients seen in the out-patient department (24.7 years vs. 23.78 years, *p* < 0.001). The mean length of stay in hospital in 2020 during the COVID19 pandemic fell from 1.45 days in 2019 to 1.28 days.

## 4. Discussion

A strength of this study was its originality. To prevent the COVID-19 infection or overcome the anxiety at a hospital level, many hospitals and healthcare organizations implemented so many policies, but the effect of these polices had not been well evaluated. We were unable to find any similarly published studies in our literature review in a literature search of the PubMed database using the following search terms: “*Covid*” *AND* “*complaints*” and “*Covid*” *AND* “*incidents*”). However, one pre-print (pre-peer review) study identified on a search on the “Google” search engine, [5] from Imperial College Healthcare NHS Trust (a group of five hospitals located in central London) found that error reporting measured from Datix, as we did in our study, significantly reduced. The authors found that in the 8 weeks following the first COVID-19 patient arriving at the trust, the number of weekly error reports consistently fell below the 52-week mean and that in 6 of the 8 weeks, the rate was more than three standard deviations below the weekly mean. Our study also found a reduction in the numbers of incidents reported, however, when corrected for the of overall patient activity, the proportions of incidents reported before and after the COVID-19 lockdown were not statistically significant.

We also found a study from Nepal published in the Lancet Global Health, [6] which found that institutional births were reduced by about 50% with an increased risk of preterm births, still births and neonatal mortality during lockdown. We however did not set out to measure these indices in our study, although the drop in births, mirrors the 18% drop in patient activity we observed in our study. This 18% drop in patient activity was as a result of a reduction in hospital admissions due to pandemic restrictions and the rationalizing of available medical resources.

We were able to identify a study [8], from the USA in the literature, which described the changing practice of nephrology during the COVID-19 pandemic. Policy and healthcare infrastructure transformed in response to the challenges of the COVID-19 pandemic, included strategies for resource management, an expansion of tele-medicine and home dialysis modalities, refined guidance on dialysis access procedures, extra measures to relieve administrative burden, and a re-evaluation of quality metrics. There was however no formal evaluation of the impact of these changes on clinical incidents, as we have undertaken in our study.

In a study from the Netherlands, metadata on weekly incident and near-incident reports from 2016 to June 2020 involving over 14,000 clients with mild to serious intellectual disability in a long-term care organisation for people with intellectual disability were subjected to interrupted time series analysis, comparing the COVID-19 period with the pre-COVID-19 period [9]. The results showed that the measures taken against COVID-19 coincided with changes in weekly incident reports and drops in incident reports were observed immediately after these measures were announced and implemented, along with an unexpected drop in medication error reports, suggesting a lower rate of incident reporting. This contrasts with the findings from our study, which did not find a significant difference in the rates of incidents reported in the immediate period after the COVID-19 lockdown period compared to a similar period the year before.

The sparsity of the literature in this field may reflect the unprecedented nature of the COVID-19 pandemic. However, it may reflect the intrinsic differences and the evolving nature of many disasters, making it challenging to develop a standardized tool for evaluating patient safety in a new disaster. Ingrassa P.L. et al. [10] tried to address this by designing an objective tool to enhance the direct evaluation of medical management during a mass casualty incident, however the tool was not applicable to the COVID-19 pandemic, which we were investigating in our study.

It has, however, been acknowledged [11] that the data were needed to understand that the quality of the care being delivered to patients during this COVID-19 pandemic have proven difficult to obtain and that, as a result, there is a lack of information that would help clinicians improve care delivery in the moment and learn for the future. The fact that we have been able to conduct our study despite this known difficulty is therefore a strength of our study. Information on clinical incidents, as we obtained in our study, is also a key component of learning systems, which is a term applied at both the system level (to describe the analysis of aggregate patient data looking for opportunities for improvement) and organizational level (to describe organizational structures, processes, and culture that promote internal learning) [12]. Boosting and expanding the learning system, specifically capturing crisis-related incidents, has been advocated as one of the quality improvement skills to deploy during the COVID-19 pandemic to support patients and organisations [13].

The proportion of incidents resulting in low or no harm in our study (98%) was also similar to the latest national figure from the national patient safety incident reports (NaPSIR) for England (97%) [14]. This provides some reassurance that, despite the outbreak of the COVID-19 pandemic, health care delivery in the NUH FH division may have remained safe. This is also reflected in the fact that the proportion of incidents reported did not decrease. The fact that the proportion of incidents reported did not decrease with the additional demands imposed by the pandemic, such that the Parliamentary and Health Service Ombudsman paused their work on existing NHS complaints and acceptance of new health complaints from 26 March 2020 to 30 June 2020, [8] was also reassuring as it suggested possible continued vigilance for patient safety in the NUH FH division.

In the designated study period, the service provided by the NUH FH division was possibly safe overall, as the majority of activities (97.9%) were not associated with any registered incidents. Furthermore, of the incidents registered, 85% resulted in no harm to patients. Our interest primarily lay in the association, or lack thereof, between incidents and changes to our new policies introduced because of the COVID-19 pandemic. We were only able to definitely link two incidents to a published policy change in FH (one to the Children’s ED admission pathway and the second to the introduction of virtual antenatal clinics). From this we deduce that measures taken to avoid COVID-19 transmission and sequelae have at large not been to the detriment of patient safety in the division.

The study is limited by the relatively short period of data collection, prompted by the rapidly changing clinical picture during the early pandemic. The period in which policies were introduced overlapped with some of period in which data were collected which was possibly too short to realize the impact of the new policies. A longer period, after introduction of new policies, may therefore provide better results. Including the demographics of the patient population in both time frames we investigated would have made it easier to make interpretable comparisons. However, going back to obtain data on the individual ages, gender and health status of each of the 71,675 patients seen in both timeframes investigated would have been a very expensive and time-consuming process, which we did not have the human and financial resources for, especially as we continued to focus on the delivery of frontline care during the pandemic. The drop in incidents, reported in 2020 compared to 2019 (672 incidents reported in 2020 compared to 826 in 2019) was most likely a result of the 18% drop in patient activity in 2020 during the initial phases of the pandemic, compared to 2019, as a result of a reduction in hospital admissions due to pandemic restrictions and the rationalizing of available medical resources, which also makes it difficult to conclude that the comparisons yielded convincing statistical results. We further investigated the possible impact of selection bias on the results by comparing data on the population and patient characteristics in both study periods. Although there was no significant difference in the proportion of patients of a male or female gender in both time periods, the mean age of patients admitted to hospital in 2020 was slightly higher compared to 2019 as was the mean age of patients seen in the out-patients. The mean length of stay in hospital in 2020 during the COVID19 pandemic also fell from 1.45 days in 2019 to 1.28 days. We therefore acknowledge that the data contained in this manuscript on their own are not sufficient to provide thorough information on the safety of the service and we would recommend that these data are collected in future studies.

Another limitation of this study was that it only described the experience from one Hospital Trust, albeit one of a large size. It may be that if we could obtain data from many hospitals, the results might be different.

Ideally, the data used to inform decisions around restoration and recovery plans could have also been gathered over a longer period to improve the quality of decision making. There are however no obvious indications that any newly implemented policies require immediate reversal. Therefore, a process of continuous monitoring and reassessment of data as we gradually transition into “regular” clinical practice appears to be safe and will help improve further, evidence-based decision making. A set of agreed-upon metrics need to be established to efficiently observe outcomes. These metrics need to be tailored to the altered style of clinical practice during the pandemic, for example virtual clinics. Decisions regarding clinical policies should ideally consider qualitative data and expert opinions. This would hopefully address some of the rigidity in our categorical outcome measures and highlight less quantifiable aspects of clinical safety during the pandemic. Methods that may be beneficial in improving our understanding include questionnaire surveys of patient experience (e.g., on virtual consultations), focus groups of staff, and other metrics required to undertake a full Quality Impact Assessment (QIA) or Equality and Quality Impact Assessment [15,16]. This includes addressing: impact on duty of quality (Care Quality Commission/ NHS constitutional standards), patient safety, clinical outcomes, patient experience, staff experience and equality and diversity.

For future reassessment and a potential complete QIA, a number of pitfalls should be addressed. Although the similar proportion of incidents reported before and after the pandemic does not suggest this, it may be that the increased pressures and demands of the pandemic reduced incident reporting, so we may not know the full impact yet. This may be addressed by updating the methods and metrics for monitoring incidents and complaints. Another limitation of this study was that certain other metrics, e.g., missed cancer diagnosis and morbidity and mortality in the community, were not captured by the methods and scope of this study. These issues require vigilance in further follow-up studies, the impact of which will unfortunately only become apparent belatedly.

A potential framework for future follow-up studies might be a mixed methods study involving multiple sites to include partners in primary care and the independent sector. The quantitative aspects of the study might involve data collection over a longer duration, for example the 12 months leading up to the COVID-19 initial lockdown, compared to the 12 months after. Initial agreement on the variables to be collected as part of the study would have to be reached and might include the demographic data of the population being investigated and broad themes into which each new policy, clinical incident and complaint would be grouped. A record will have to be kept of the timeframes over which the policies were introduced. The qualitative aspects of the study might involve focus groups and questionnaire surveys of staff and patients with adequate representation from the regions involved and the site of encounter (primary care, secondary care or the independent sector).

Each clinical incident reported during the study period will have to be read and linked to a broad theme into which new clinical policies have been grouped and whether or not it was related to COVID-19. Quantitative data analysis will ideally be carried out by investigators blind to the allocation of clinical incident to themes and time frames. The primary outcome measure might be the rates of clinical incidents reported in the 12 months leading up to the COVID-19 initial lockdown, compared to the 12 months after. Secondary outcome measures would include the rates of COVID-19-related incidents in clinical incidents linked to a new policy compared to the rates in clinical incidents not linked to a new policy. It will also be important to investigate whether or not there was a significant increase in incidents linked to one policy theme compared to others and compared to the previous year. A timeframe analysis might also support any associations identified. Qualitative analysis of the data obtained from the focus groups and free text responses in the questionnaire survey might involve a thematic analysis of the transcripts of the interviews and free text survey responses using a software program, for example nVivo, to identify themes related to the introduction of new clinical policies and clinical incidents and the COVID-19 pandemic.

## 5. Conclusions

New policies introduced in the NUH FH division, during the COVID-19 pandemic, were associated with similar rates of reported clinical incidents, when compared with the previous year. There was no difference in the proportion of incidents reported on Datix^®^ just after the COVID-19 lockdown in 2020, compared to a similar period in 2019. There was also no statistically significant difference found in the proportion of incidents classed as resulting in serious incidents or low or no harm. At the stage of submission of this article, we were able to link only two COVID-19 associated incidents with a new policy in response to the COVID-19 pandemic, in the NUH FH division: one to the Children’s ED admission pathway and the second to the introduction of virtual antenatal clinics. There were, however, only 18 complaints related to COVID-19, out of 32,237 patient activities, reported through our PALS, which may not have provided sufficient information to explain patient satisfaction. It therefore makes it challenging to confidently conclude that the NUH FH continued to provide a safe service overall.

Based on this initial assessment however, we consider it safe, with a degree of caution, to extend the pathways and procedures introduced in response to COVID-19, without risk of significant detriment to patient safety/experience in FH. Whether it be in the midst of a pandemic or not, a serious incident in a 2-month period is still a figure we should strive to reduce. By initiating a longer-term follow-up process investigating our procedures and pathways, with improved metrics and data collection, we hope to minimize the additional impact caused by the COVID-19 pandemic in Family Health at NUH. We also hope that this study provides a useful framework for conducting similar studies in other settings to determine the national/international impact of the COVID-19 pandemic on overall patient safety.

## Figures and Tables

**Table 1:** Incident categories with statistically significant increases between COVID-19-related and non-COVID-19-related incidents in the period (23 March 2020 to 29 May 2020).

Category	Number of COVID-19-Related Incidents 2020 (*n* = 61)	Number of Non-COVID-19-Related Incidents (*n* = 611)	Percentage of (COVID-19-Related Incidents 2020)	Percentage of Non-COVID-19-Related Incidents	Percentage Difference. COVID 19 vs. Non-COVID 19-Related Incidents	*p* Values
Child Death	2	2	3.3%	0.3%	3.0%	0.0472
Delay/failure to treatment or procedure	11	47	18.0%	7.7%	10.7%	0.0123
Information Governance	7	19	11.5%	3.1%	8.6%	0.0039

**Table 2:** Association between complaints and pathway or procedural change in Nottingham University Hospital (NUH) Family Health (FH) division.

Specialty	Subjects	Was the Complaint Obviously Associated with a Published Pathway or Procedural Change in the Family Health Division?
Obstetrics	Safety	No
Gynaecology	Communication	No
Children and Young People	Safety	No
Obstetrics	Clinical Treatment	No
Obstetrics	Clinical Treatment	Uncertain
Obstetrics	Communication	Uncertain
Obstetrics	Clinical Treatment	Uncertain
Obstetrics	Safety	No
Fertility Clinic (Andrology)	Appointments	No
Children and Young People	Communication	No
Obstetrics	Safety	No
Obstetrics	Safety	No
Children and Young People	Communication	No
Obstetrics	Clinical Treatment	Uncertain
Obstetrics	Appointments	Uncertain
Gynaecology	Appointments	Uncertain
Children and Young People	Clinical Treatment	No
Obstetrics	Clinical Treatment	No

**Table 3:** Population and Patient Characteristics NUH Family Health division in 2019 compared with 2020.

Population and Patient Characteristics	2019	2020	*p* Values
Total number (%) of in-patient appointments	9022 (23%)	6791 (21%)	<0.0001
Total number (%) of out-patient appointments	29495 (77%)	25225 (79%)	<0.0001
Total number (%) of new outpatient appointments (e.g., clinics)	8841 (30%)	6951 (28%)	<0.0001
Total number of follow up outpatient appointments (e.g., clinics)	20654 (70%)	18274 (72%)	<0.0001
total number (%) of male patients seen	10,606 (27.54%)	9002 (28.1%)	Ns
total number (%) of female patients seen	27,903 (72.44%)	23,003 (79.9%)	Ns
total (%) unknown gender seen	8 (0.02%)	11 (0.03%)	Ns
Mean (+/− SD) age of inpatients seen in FH division (years)	17.2 (15.2)	18.9 (14.78)	<0.0001
Mean (+/− SD) age of out-patients seen in FH division (years)	23.78 (16.93)	24.7 (18.24)	<0.0001
Mean (+/− SD) length of stay in the hospital for in-patients (days)	1.45 (2.78)	1.288 (2.7)	0.0002

Ns = Not significant; FH = Family Health.

## Data Availability

The datasets used and/or analysed during the current study are available from the corresponding author on reasonable request.

## Abbreviations

QIA	Quality Impact Assessment
COVID-19	Coronavirus disease 2019
NHS	National Health Service
NUH	Nottingham University Hospital
FH	Family Health
PALS	Patient Advice and Liaison Service

## References

[1] Zhu N., Zhang D., Wang W., Li X., Yang B., Song J., Zhao X., Huang B., Shi W., Lu R. (2019). A novel coronavirus from patients with pneumonia in China. N. Engl. J. Med..

[2] Coronavirus Reported Cases and Deaths by Country, Territory, or Conveyance. https://www.worldometers.info/coronavirus/.

[3] Wikipedia Contributors Timeline of the COVID-19 Pandemic in England. https://en.wikipedia.org/w/index.php?title=Timeline_of_the_COVID-19_pandemic_in_England&oldid=977185365.

[4] Gilbert A.W., Billany J.C.T., Adam R., Martin L., Tobin R., Bagdai S., Galvin N., Farr I., Allain A., Davies L. (2020). Rapid implementation of virtual clinics due to COVID-19: Report and early evaluation of a quality improvement initiative. BMJ Open Qual..

[5] Denning M., Goh E.T., Scott A., Martin G., Markar S., Flott K., Mason S., Almonte M., Clarke J., Beatty W. (2020). What has been the impact of Covid-19 on safety culture? A case study from a large metropolitan teaching hospital. medRxiv.

[6] Kc A., Gurung R., Kinney M.V., Sunny A., Moin M., Basnet O., Paudel P., Bhattarai B., Subedi K., Lawn J.E. (2020). Effect of the COVID-19 pandemic response on intrapartum care, stillbirth, and neonatal mortality outcomes in Nepal: A prospective observational study. Lancet Glob. Health.

[7] Parliamentary and Health Service Ombudsman Coronavirus: Information for Complaint Handling Teams. https://www.ombudsman.org.uk/organisations-we-investigate/coronavirus-information-complaint-handling-teams.

[8] Truong T., Dittmar M., Ghaffari A., Lin E. (2020). Policy and pandemic: The changing practice of nephrology during the coronavirus disease—2019 outbreak. Adv. Chronic Kidney Dis..

[9] Huengel C., Tummers J., Embregts P.J.C.M., Leusekin G.L. (2020). Impact of the initial response to COVID-19 on long-term care for people with intellectual disability: An interrupted time series analysis of incident reports. J. Intellect. Disabil. Res..

[10] Ingrassia P.L., Prato F., Geddo A., Colombo D., Tengattini M., Calligaro S., La Mura F., Franc J.M., Della Corte F. (2010). Evaluation of medical management during a mass casualty incident exercise: An objective assessment tool to enhance direct observation. J. Emerg. Med..

[11] Austin J.M., Kachalia A. (2020). The state of health care quality measurement in the era of COVID-19: The importance of doing better. JAMA.

[12] Bohmer R., Shand J., Allwood D., Wragg A., Mountford J. (2020). Learning systems: Managing uncertainty in the new normal of Covid-19. NEJM Catal. Innov. Care Deliv..

[13] Staines A., Amalberti R., Berwick D.M., Braithwaite J., Lachman P., Vincent C.A. (2021). COVID-19: Patient safety and quality improvement skills to deploy during the surge. Int. J. Qual. Health Care.

[14] NHS England, NHS Improvement (2020). NRLS National Patient Safety Incident Reports: Commentary. https://improvement.nhs.uk/documents/6571/NAPSIR_commentary_March_2020.pdf.

[15] National Quality Board (2012). How to: Quality Impact Assess Provider Cost Improvement Plans. https://assets.publishing.service.gov.uk/government/uploads/system/uploads/attachment_data/file/212819/How-to-Quality-Impact-Assess-Provider-Cost-Improvement-Plans-.pdf.

[16] NHS Improvement (2018). Developing Workforce Safeguards. https://improvement.nhs.uk/documents/3320/Developing_workforce_safeguards.pdf.

